# A Self-Calibration Method for Static and Dynamic Near-Bit Well Inclination Measurement

**DOI:** 10.3390/s26216782

**Published:** 2026-10-27

**Authors:** Naipeng Liu, Shugang Hou, Zhen Yang, Lixia Tao, Yonghua Li, Jieran Zhu, Jinlong Dong, Yule Hu

**Affiliations:** 1 Geosteering & Logging Research Institute, Sinopec Matrix Corporation, Qingdao 266000, China; 2 Sinopec Key Laboratory of Measurement and Control While Drilling, Qingdao 266000, China; 3 School of Petroleum and Chemical Engineering, Dongying Vocational College, Dongying 257091, China; 4 Faculty of Engineering, China University of Geosciences, Wuhan 430074, China

**Keywords:** near-bit inclination, installation errors, dynamic inclination, multivariate linear regression

## Abstract

With the depletion of oil and gas resources and increasing exploration difficulty, dynamic well inclination measurement has become increasingly important. Near-bit dynamic well inclination measurement can improve borehole trajectory calculation accuracy and drilling efficiency. However, when the well inclination measurement module is installed eccentrically, installation errors are easily introduced. Additionally, during drill collar rotation, all three-axis accelerations are affected by centrifugal acceleration. These factors collectively impact the accuracy of well inclination measurement. Current dynamic well inclination measurement methods primarily focus on extracting gravitational acceleration while often neglecting the influence of installation errors on gravitational acceleration. In this paper, a triaxial-acceleration well inclination measurement module based on a single quartz flexure accelerometer and a dual-axis MEMS accelerometer is designed. The scale factor, zero bias, and non-orthogonal angle of the triaxial acceleration are calibrated using a hybrid ellipsoid and Levenberg–Marquardt method. A self-calibration algorithm for static and dynamic well inclination measurements based on multivariate linear regression is proposed, which simultaneously solves for installation errors and actual well inclination. The installation-error calibration was independently verified against a calibrated standard high-precision three-axis turntable (±0.01°), with an inclination error below 0.1° at nominal inclinations ranging from 0° to 90°. In the rotational tests, covering inclinations ranging from 5.36° to 89.64° and rotational speeds in the range of 30–150 r/min, the well inclination error is within ±0.18°. Field test results show that the near-bit inclination follows the same trend as the MWD survey, with a mean absolute error of about 0.2°.

## 1. Introduction

High-precision well inclination measurement is the basic guarantee for high-quality borehole trajectory control, which can provide support for the accurate exploration of resource reserves and efficient resource development. Conventional drilling instruments are usually installed far away from the drill bit, which cannot reflect the drilling trajectory of the drill bit and the information of strata encountered in a timely manner. Moreover, the current drilling measurement is usually static and requires stopping drilling, which affects drilling efficiency. Meanwhile, frequent stopping of drilling measurements will affect the stability of the well wall, which has an impact on drilling safety. To control the borehole trajectory more accurately, near-bit dynamic inclination measurement has become the development trend of directional drilling measurement [1,2,3].

Current well inclination dynamics measurements are mainly considered in terms of removing shock noise, vibration noise, and centrifugal acceleration to extract interference-free gravity acceleration. For shock noise removal, limiting filtering is usually used. For vibration noise removal, initially, scholars used low-pass filter removal, such as the Butterworth low-pass filter [4] and FIR filter [5], but it is difficult to filter out the noise in the passband, and subsequently scholars have used the Kalman filter [6,7] unscented Kalman filter [8,9,10,11,12,13,14], improved unscented Kalman filter, minimum mean square error adaptive filtering algorithm [3], heavy tail measurement filter [15], and other filters to filter out vibration noise, and achieved good results.

For the removal of centrifugal acceleration, some scholars use mirror symmetric accelerometers to eliminate centrifugal acceleration [16,17,18], but mirror symmetric installation has errors, and it is difficult to eliminate centrifugal acceleration. Because the magnetic sensor is virtually immune to interference from complex motions at the bottom of the well, some scholars have used cross-correlation extraction to extract gravitational acceleration based on magnetic signal [19]. By combining the abovementioned algorithms, removing vibration noise and centrifugal acceleration, undisturbed gravitational acceleration can be derived and well inclination can be calculated.

Although the current study removes vibration noise and centrifugal acceleration, it ignores the influence of installation error. During equipment assembly, storage, and transportation, the accelerometer’s coordinate system and the drill collar’s coordinate system can deviate from each other and not coincide, thus affecting static and dynamic well inclination measurements. The X-, Y-, and Z-axis acceleration components are affected by centrifugal acceleration due to installation errors, and current research on eliminating centrifugal acceleration focuses on the X and Y axes while neglecting the Z axis. For the calibration of accelerometer installation error, Deng et al. [20] established a mapping relationship between the actual installation coordinate system of the inclinometer module and the borehole coordinate system for vertical-drilling wells and derived a functional relationship for calculating the real well inclination by using the actual output signals of the inclinometer module, which realizes the calibration of the installation error of the inclinometer module. However, there is a lack of research on the impact of installation error on dynamic well inclination measurement.

In this paper, from the perspective of the calibration of installation error, a self-calibration measurement method for static and dynamic well inclinations is proposed. The method judges the measurement state based on the acceleration variance. When in the rotational condition, the dynamic well inclination and installation error are solved by multivariate linear regression, and the installation error is stored. In the static condition, the installation error is read to solve for the static well inclination. This updates the installation errors once rotation occurs, thereby addressing the problem of installation-error changes during storage, transportation, and use.

The main novelty of this work lies in the simultaneous estimation of the well inclination and the installation errors through multivariate linear regression. Unlike conventional procedures in which installation errors are calibrated separately before the dynamic measurement, the proposed method estimates both quantities from the same regression, using the natural rotational excitation of the drill collar. This removes the need for a separate installation-error calibration, makes the method insensitive to the centrifugal term $\omega^{2}r$, and allows the installation errors to be updated online during rotation.

## 2. Methods

### 2.1. Circuit Design for Well Inclination Measurement

**Figure 1:**
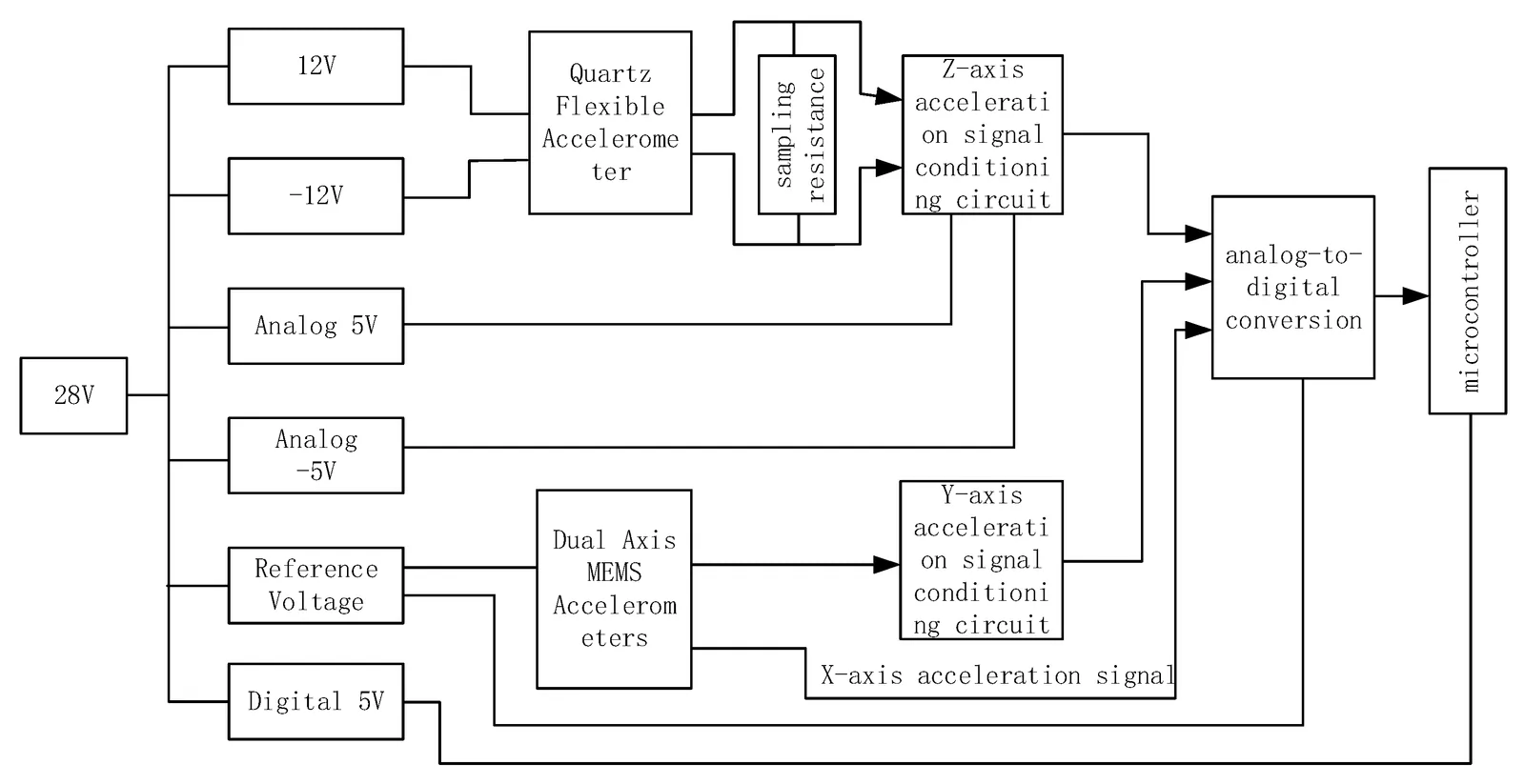
Block diagram of well inclination measurement circuit design.

Current acceleration measurement sensors are mainly divided into two kinds: quartz flexure accelerometer and MEMS accelerometer. The quartz flexure accelerometer has high accuracy but a high cost and large size; the MEMS accelerometer has the advantages of low cost, small size, and good shock resistance, but there are disadvantages such as large temperature nonlinearity and time drift. Considering the cost, size, and accuracy, this paper is designed to measure well inclination using a single quartz flexure accelerometer for the Z axis and a dual-axis MEMS accelerometer for the X and Y axes. This solution not only meets the needs of high-precision well inclination measurement but also the requirements of low cost and small size. The block diagram of the circuit design is shown in Figure 1: the power supply is 28 V, which is converted to ±12 V for the quartz flexure accelerometer power supply, analog ±5 V for the signal conditioning circuit power supply, reference voltage for the dual-axis MEMS accelerometer power supply and analog-to-digital conversion reference voltage, and digital 5 V for the power supply of microprocessor and communication circuits. The output of the quartz flexure accelerometer is a positive and negative current output. It is adjusted to a positive voltage by a sampling resistor and the signal conditioning circuit for sampling by the analog-to-digital converter with a range of ±4*g*. The range of the dual-axis MEMS accelerometer is ±8*g*. X-axis acceleration is affected by centrifugal acceleration during the rotation process, and thus the X-axis acceleration range is not altered. The Y axis is aligned with the radial direction in the chosen geometry, so it is not affected by centrifugal acceleration ($\omega^{2}r$). However, it can be affected by tangential acceleration (r dω/dt) during speed transients. Y-axis acceleration is therefore adjusted to ±5*g* by the signal conditioning circuit to improve the measurement accuracy.

### 2.2. Calibration Based on Hybrid Ellipsoid and Levenberg–Marquardt Method

The designed well inclination measurement module needs to be calibrated for its scale factor, zero bias, and nonorthogonal error [21]. The relationship between calibration acceleration and voltage is shown in Equation (1)

(1) $$\left[\begin{array}{c} G_{ax}\\ G_{ay}\\ G_{az} \end{array}\right]=\left[\begin{array}{ccc} K_{xx} & K_{xy} & K_{xz}\\ K_{yx} & K_{yy} & K_{yz}\\ K_{zx} & K_{zy} & K_{zz} \end{array}\right]\left[\begin{array}{c} A_{x}-B_{x}\\ A_{y}-B_{y}\\ A_{z}-B_{z} \end{array}\right]$$

*G_ax_*, *G_ay_*, and *G_az_* are the calibrated triaxial-acceleration components; *K_xx_*, *K_yy_*, and *K_zz_*  are the scale factors; *K_xy_*, *K_xz_*, *K_yx_*, *K_yz_*, *K_zx_*, and *K_zy_* are the nonorthogonal errors; *A_x_*, *A_y_*, and *A_z_* are the triaxial-acceleration measurement voltages; and *B_x_*, *B_y_*, and *B_z_* are the zero biases.

The main calibration methods include the six-position method, the twenty-four-position method, the ellipsoid method, and the Levenberg–Marquardt (LM) method [22]. The six-position method has a low calibration accuracy; the twenty-four-position method requires a high-accuracy inclinometer calibration turntable. The ellipsoid and LM methods are based on the vector sum of triaxial acceleration that is 1*g* (the gravitational acceleration), which is shown in

(2) $$\sqrt{{G_{ax}}^{2}+{G_{ay}}^{2}+{G_{az}}^{2}}=1g$$

This requires low accuracy of the inclinometer calibration turntable and reduces calibration costs. The ellipsoid method can only calibrate the scale factor and zero bias, while the LM method requires high initial values, and the larger the deviation of the initial values, the lower the solution efficiency. To improve calibration efficiency, this paper adopts a hybrid calibration method of the ellipsoid and LM methods. First, the triaxial acceleration at different well inclination and tool-face angle positions was measured, then the scale factor and zero bias were initially solved by the ellipsoid method, and then the scale factor, the zero bias, and the nonorthogonal angle were further solved by the LM method.

### 2.3. Static and Dynamic Well Inclination Self-Calibration Method

**Figure 2:**
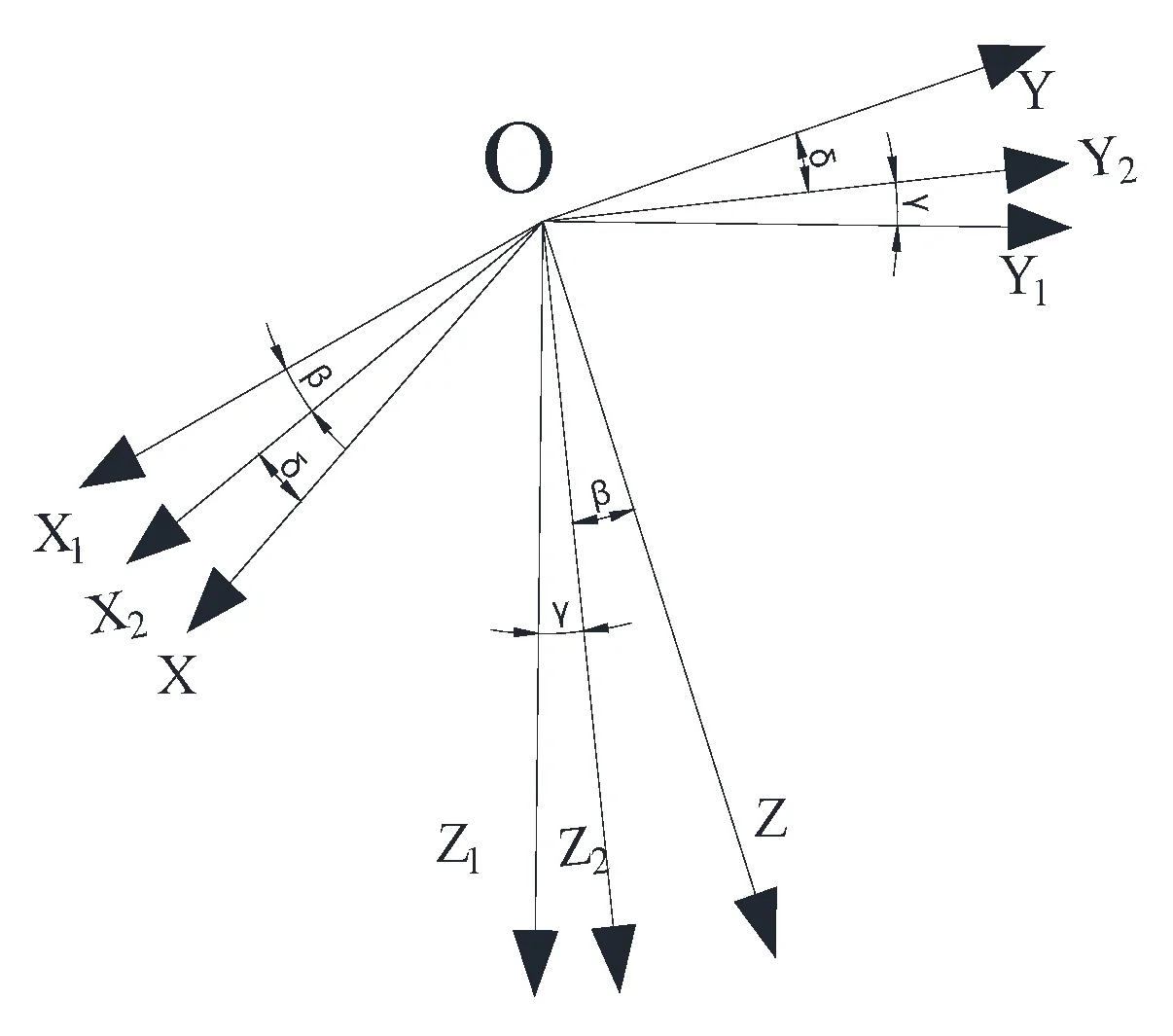
Schematic diagram of accelerometer coordinate system transformed into the drill collar coordinate system.

After the accelerometer is calibrated, an installation error occurs when it is installed on the drill collar, which is the coordinate system of the accelerometer that does not coincide with the coordinate system of the drill collar, so the installation error needs to be calibrated. The coordinate system of the accelerometer can be transformed into the coordinate system of the drill collar by the Euler transformation, as shown in Figure 2. X_1_Y_1_Z_1_ is the coordinate system of the accelerometer installation, rotate angle *γ* around the X_1_ axis to get the X_1_Y_2_Z_2_ coordinate system, rotate angle *β* around the Y_2_ axis to get the X_2_Y_2_Z coordinate system, and rotate *δ* around the Z axis to get the XYZ coordinate system, which is the coordinate system of the drill collar. The coordinate conversion formula is shown in Equation (3).

(3) $$\left[\begin{array}{c} G_{\mathrm{bx}}\\ G_{\mathrm{by}}\\ G_{\mathrm{bz}} \end{array}\right]=S\left[\begin{array}{c} G_{\mathrm{ax}}\\ G_{\mathrm{ay}}\\ G_{\mathrm{az}} \end{array}\right]$$

where *G_bx_*, *G_by_*, and *G_bz_* are triaxial-acceleration components of the drill collar coordinate system; ***S*** is shown in Equation (4):

(4) $$S=\left[\begin{array}{ccc} cos\delta cos\beta & -sin\delta cos\gamma -sin\gamma sin\beta cos\delta & sin\delta sin\gamma -cos\delta cos\gamma sin\beta \\ sin\delta cos\beta & cos\delta cos\gamma -sin\beta sin\delta sin\gamma & -sin\gamma cos\delta -sin\beta sin\delta cos\gamma \\ sin\beta & sin\gamma cos\beta & cos\gamma cos\beta \end{array}\right]$$

**Figure 3:**
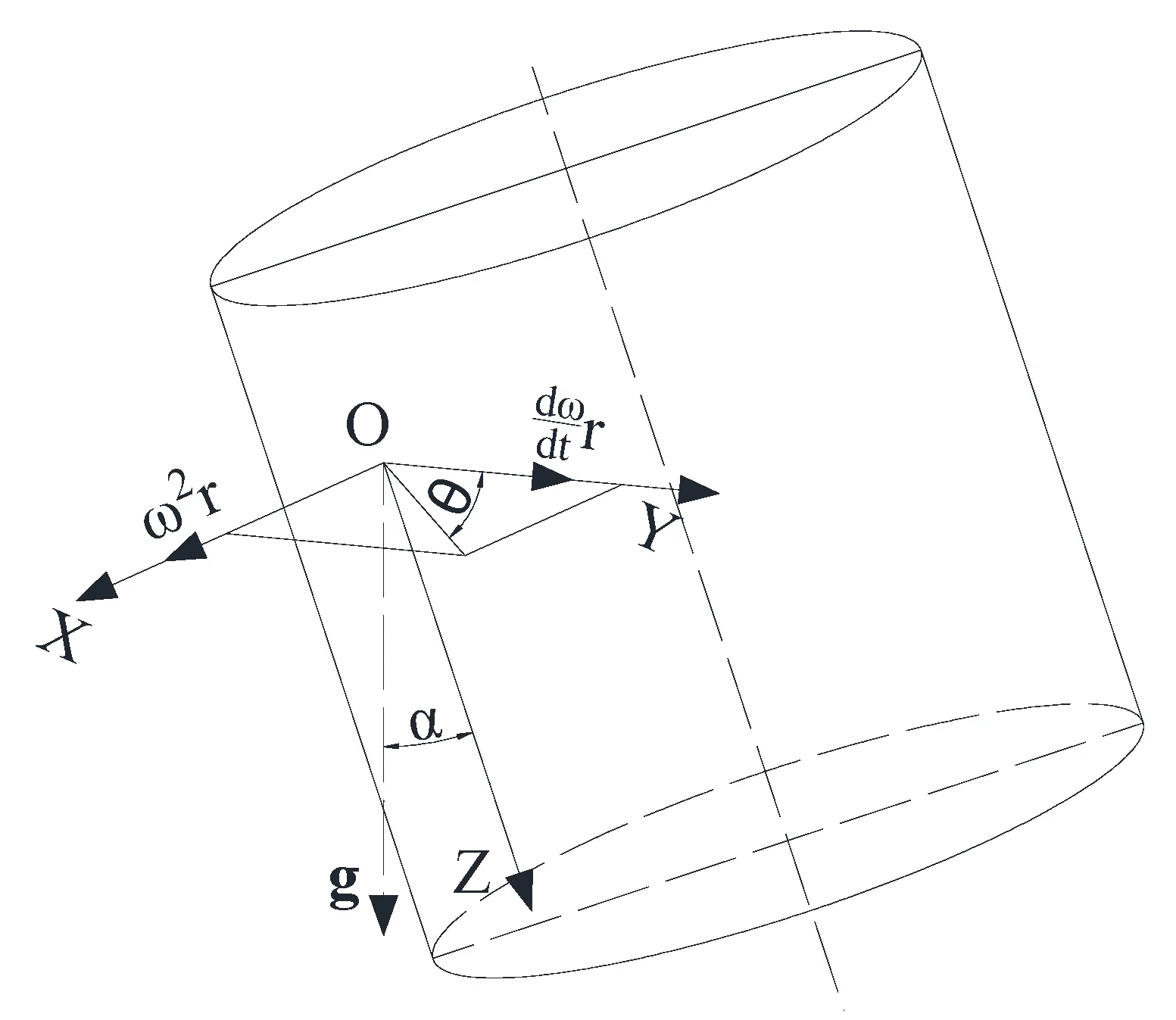
Schematic diagram of accelerometer coordinate system transformed to drill collar coordinate system.

The following assumptions are made in the derivation of Equation (5). The drill collar rotates about a fixed axis; the eccentric distance r between the accelerometer and the drill collar axis is constant within a regression window; axial and lateral vibrations are treated as zero-mean noise and are suppressed by the Kalman filter and the regression; and the angular acceleration dω/dt is nonzero only during speed transients. During drilling, the centrifugal force generated by the rotation of the drill collar interferes with the triaxial-acceleration measurement, and the triaxial-acceleration component during rotation is shown in Figure 3. The relationship between the three-axis acceleration, the well inclination *α*, the tool-face angle *θ*, and the rotational speed *ω* is shown in Equation (5).

(5) $$\left[\begin{array}{c} G_{bx}\\ G_{by}\\ G_{bz} \end{array}\right]=\left[\begin{array}{c} gsin\alpha sin\theta \\ gsin\alpha cos\theta \\ gcos\alpha \end{array}\right]+\left[\begin{array}{c} \omega^{2}r\\ \frac{d\omega }{dt}r\\ 0 \end{array}\right]$$

where g is the gravitational acceleration, r is the distance between the accelerometer and the center axis of the drill collar, and dω/dt is the angular acceleration, which is nonzero only during speed transients. The term $\omega^{2}r$ represents the centrifugal acceleration, while the term r dω/dt represents the tangential acceleration. Let the installation-error matrix ***S*** be:

(6) $$S=\left[\begin{array}{ccc} s_{11} & s_{12} & s_{13}\\ s_{21} & s_{22} & s_{23}\\ s_{31} & s_{32} & s_{33} \end{array}\right]$$

From (4)–(6), we can get:

(7) $$\mathrm{gsin}\alpha \sin\theta +\omega^{2}r=s_{11}g_{aX}+s_{12}g_{\mathrm{aY}}+s_{13}g_{\mathrm{aZ}}$$

(8) $$\mathrm{gsin}\alpha \cos\theta +\frac{d\omega }{\mathrm{dt}}r=s_{21}g_{aX}+s_{22}g_{\mathrm{aY}}+s_{23}g_{\mathrm{aZ}}$$

(9) $$\mathrm{gcos}\alpha =s_{31}g_{aX}+s_{32}g_{\mathrm{aY}}+s_{33}g_{\mathrm{aZ}}$$

(10) $$s_{31}^{2}+s_{32}^{2}+s_{33}^{2}=1$$

The multivariate linear regression is formulated as a least-squares problem:

$${min}_{c}∥Xc-y{∥}_{2}^{2},$$

where X is the design matrix containing the sinusoidal terms in the tool-face angle, y is the measured acceleration vector, and c is the coefficient vector. The solution is obtained in closed form as $c={\left(X^{⊤}X\right)}^{-1}X^{⊤}y$. The installation angles were constrained to physically plausible ranges. The well inclination α and the installation-error angles β and γ were then recovered from the fitted coefficients through Equations (9) and (10) and the inverse transformation of Equation (4).

The regression window is one complete rotation cycle, with a minimum of 1000 samples. For identifiability, the tool-face coverage within the window must span at least 270°, so that the installation angles can be decoupled from the inclination. If the tool-face coverage is partial, the design matrix becomes ill-conditioned and the estimates of β and γ become unstable. Near-vertical inclinations have larger condition numbers because the horizontal acceleration component is small, but the solution remains acceptable when the tool-face span exceeds 270°. These conditions were verified in the experiments reported in Section 3.

Therefore, we can establish the multivariate fitted linear regression equation of triaxial acceleration by (9), and then solve the well inclination angle α and installation-error angles *β*, *γ* according to Equations (4) and (10). Because the installation-error angle *δ* rotational plane is perpendicular to the Z axis, it does not affect the well inclination calculation, and we can assume *δ* is 0. From the solution method, it can be seen that the model can ignore the effect of centrifugal acceleration *ω*^2^*r*. Acceleration usually contains a lot of noise during the rotation of the drill collar, and the Kalman filter is commonly used [23] to eliminate acceleration noise. So, the Kalman filter was used to filter out vibration noise in this study.

A constant-acceleration Kalman filter was used for denoising. The state vector is $x={\left[G,\dot{G}\right]}^{⊤}$ and the process model is

$$F=\left[\begin{array}{cc} 1 & \Delta t\\ 0 & 1 \end{array}\right],$$

where Δt is the sampling interval. The process-noise covariance is $Q=diag\left(q_{1},q_{2}\right)$, where $q_{1}=1\times 10^{-6}\;g^{2}$ and $q_{2}=1\times 10^{-4}\;g^{2}/s^{2}$; the measurement-noise covariance is $R=1\times 10^{-6}\;g^{2}$. The filter was initialized from the first 0.5 s of static data, where $x_{0}={\left[\bar{G},0\right]}^{⊤}$ and $P_{0}=diag\left(10^{-4},10^{-2}\right)$.

The measurement state was classified as rotational when the acceleration variance exceeded $\sigma_{th}^{2}=5\times 10^{-4}\;g^{2}$ and the rotational speed exceeded $\omega_{th}=20\;r/min$; otherwise, it was classified as static. The acceleration variance was computed over a 0.5 s sliding window (250 samples at 500 Hz). The regression window is one complete rotation cycle with a minimum of 1000 samples. The installation-error parameters were updated once per complete rotation and stored in the firmware.

According to the abovementioned theories, we designed the algorithm (Algorithm 1) to realize static and dynamic well inclination self-calibrations. First, the calibration matrix ***K*** and zero bias ***B*** were derived by calibration and initialized, collecting acceleration at high frequency for a period of time. The triaxial-acceleration components in the accelerometer coordinates were calculated and acceleration was denoised using the Kalman filter. Then, we obtained the variance of the acquired acceleration to determine the rotational state. When the variance was greater than the threshold value, it was judged to be the rotational condition. At this time, the multivariate linear fitting triaxial acceleration was used to solve for the well inclination *α* and the installation error angles *γ*, *β*, and the installation error was stored to be used for the calculation of well inclination under the static state. When the variance was less than the threshold value, it was judged to be the static condition. At this time, the installation-error angles *γ*, *β* were read; then the installation-error matrix ***S*** was calculated, and the static well inclination was solved. The algorithm calculates well inclination under rotational conditions and stores the installation error for static well inclination calculations, realizing self-calibrated dynamic and static well inclination measurements, thus solving the problem of installation error.

The initialization and recalibration procedure was as follows. On power-up, the system loaded the last stored installation-error parameters from the firmware. If no valid rotational calibration has been performed since the last reinstallation, the stored values may be outdated. In this case, the system flags the condition and requires at least one complete rotation before reporting a calibrated static inclination. Therefore, the method requires at least one new rotational period for the installation errors to be updated.
**Algorithm 1**: Static and dynamic well inclination self-calibration**Input:**Calibration matrix $K=\left[\begin{array}{ccc} K_{xx} & K_{xy} & K_{xz}\\ K_{yx} & K_{yy} & K_{yz}\\ K_{zx} & K_{zy} & K_{zz} \end{array}\right]$; Zero bias $B=\left[\begin{array}{c} B_{x}\\ B_{y}\\ B_{z} \end{array}\right]$; Voltage dataset of accelerometer $A_{i}=\left[\begin{array}{c} A_{xi}\\ A_{yi}\\ A_{zi} \end{array}\right],\;i=1,2,\ldots ,m$.1:**for** i=1,2,…,m **do**2:
$G_{ai}=K\left(A_{i}-B\right)$;3:
$G_{ai,kalman}=Kalman\left(G_{ai}\right)$;4:**end for**5:$G_{ax,average}=\frac{1}{m}\sum_{i=1}^{k}G_{axi,kalman}$;6:$G_{ay,average}=\frac{1}{m}\sum_{i=1}^{k}G_{ayi,kalman}$;7:$G_{az,average}=\frac{1}{m}\sum_{i=1}^{k}G_{azi,kalman}$;8:$G_{ax,variance}=\frac{1}{m}\sum_{i=1}^{k}(\left.G_{axi,kalman}-G_{ax,average}{\left.)\right.}^{2}\right.$;9:**if** $G_{ax,variance}>\sigma_{th}^{2}$ **and** $\omega >\omega_{th}$ **then**10:
Multivariate linear regression and solve α, β, γ;11:
Store installation error angle β, γ to flash;12:**Else**13:
Read the installation error angle β, γ from flash;14:
$S=\left[\begin{array}{ccc} \cos\beta & -\sin\gamma \sin\beta & -\cos\gamma \sin\beta \\ 0 & \cos\gamma & -\sin\gamma \\ \sin\beta & \sin\gamma \cos\beta & \cos\gamma \cos\beta \end{array}\right]$;15:
$\left[\begin{array}{c} G_{bx}\\ G_{by}\\ G_{bz} \end{array}\right]=S\left[\begin{array}{c} G_{ax,average}\\ G_{ay,average}\\ G_{az,average} \end{array}\right]$16:
$\alpha =\arctan\frac{\sqrt{G_{bx}^{2}+G_{by}^{2}}}{G_{bz}}$;17:**end if****Output:**Well inclination: α.

## 3. Results

### 3.1. Calibration Based on Hybrid Ellipsoid and Levenberg–Marquardt Method

**Figure 4:**
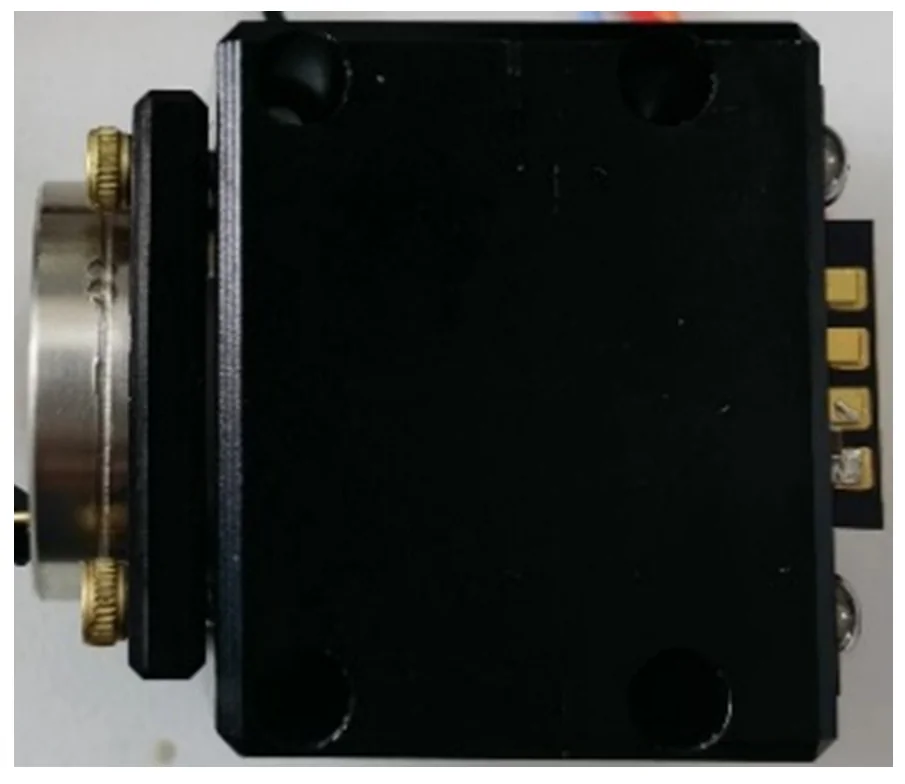
Triaxial-acceleration sensor assembly diagram.

The triaxial-acceleration sensor is assembled by a combination of a single-axis quartz flexure accelerometer and a dual-axis MEMS accelerometer, as shown in Figure 4. The triaxial-acceleration voltages were measured on an inclinometer calibration station for eighteen different well inclinations and tool-face angles, and the results are shown in Table 1.

**Figure 5:**
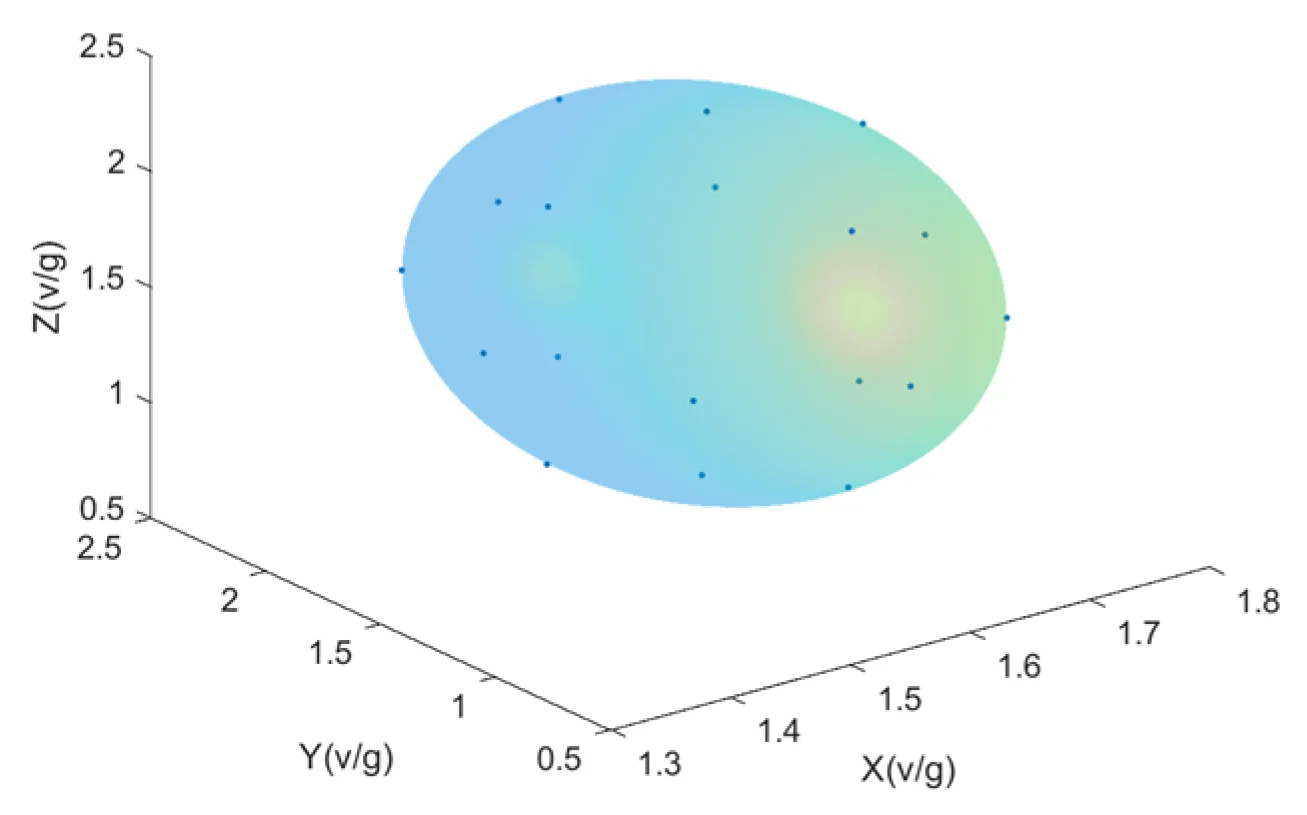
Ellipsoid fitting results for triaxial acceleration.

The ellipsoid method was first used to initially solve for the scale factor and zero bias, and the ellipsoid fitting results are shown in Figure 5.

**Figure 6:**
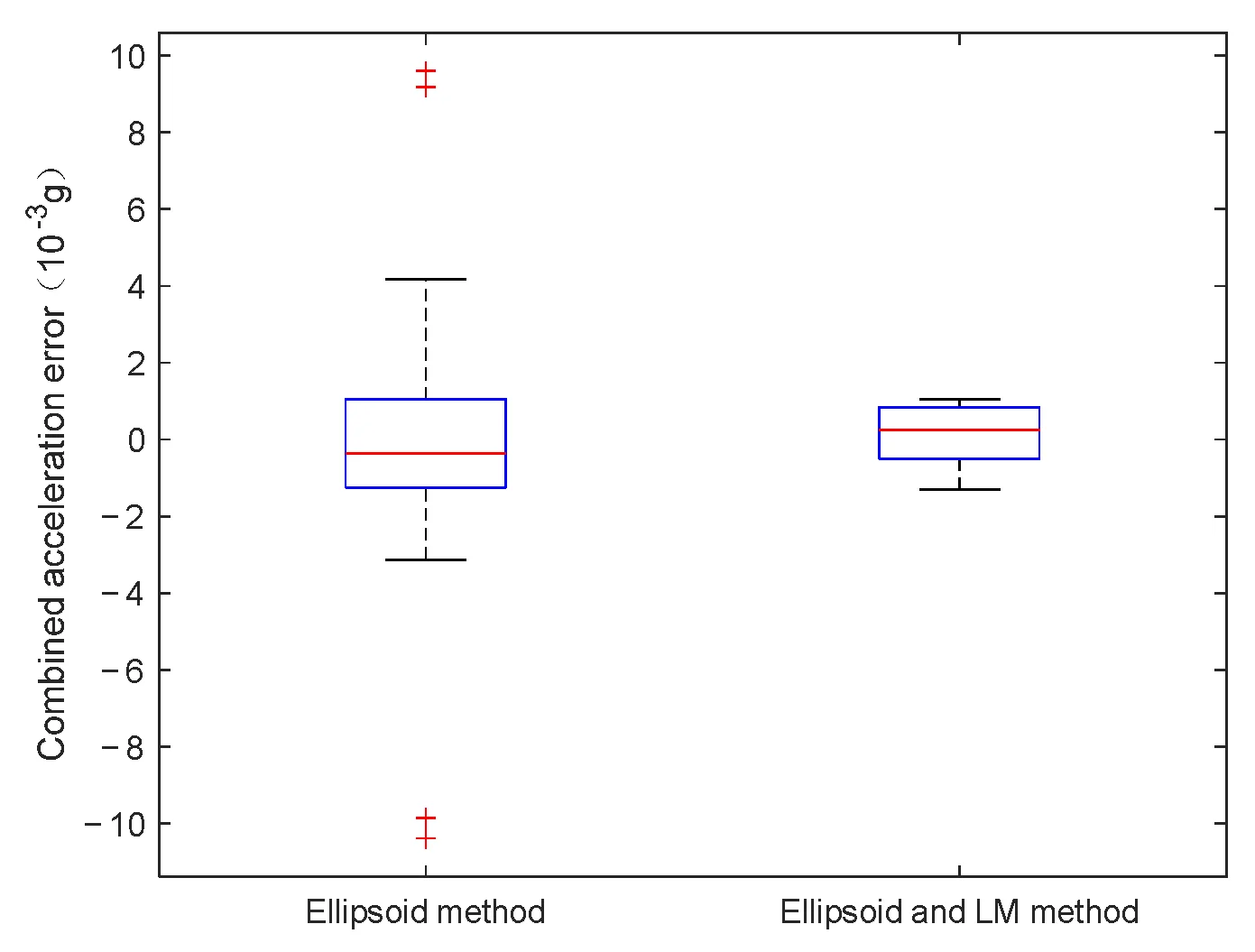
Comparison of errors between ellipsoid and LM methods.

Then, the LM method is used to further calibrate the scale factor, zero bias, and nonorthogonal angle. The parameters calibrated by the LM and ellipsoid methods are shown in Table 2, and the comparison of the error of the three-axis combined acceleration is shown in Figure 6. The sum of three-axis acceleration vectors calibrated by the LM method has an error of less than 2 × 10^−3^ g, which is more effective. To quantify the improvement of the hybrid method, the calibration errors of the ellipsoid method alone and the proposed hybrid ellipsoid–LM method are compared in Table 3. The ellipsoid method alone has an RMSE of $5.04\times 10^{-3}$ g and a maximum absolute error of $10.39\times 10^{-3}$ g, whereas the hybrid method reduces the RMSE to $0.68\times 10^{-3}$ g and the maximum absolute error to $1.31\times 10^{-3}$ g. This corresponds to a reduction of about 86.5% in RMSE and 87.4% in maximum error. The hybrid method therefore significantly improves the calibration accuracy compared with the ellipsoid method alone.

### 3.2. Installation-Error Calibration and Independent Verification

**Figure 7:**
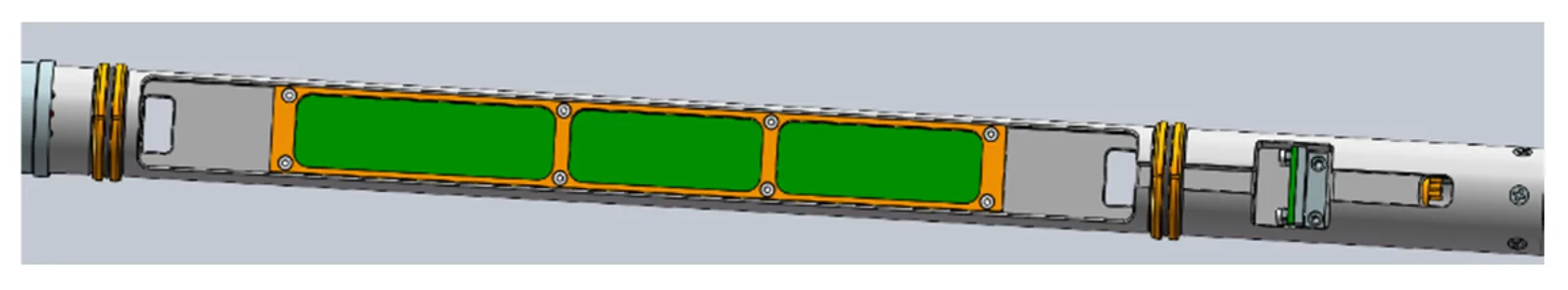
Mechanical design of the probe. The triaxial-acceleration sensing module is mounted at the middle of the probe.

After accelerometer calibration, the sensors are assembled inside a single probe (sonde). Figure 7 shows the mechanical design of the probe, in which the triaxial-acceleration sensing module is mounted at the middle of the probe, inside the drill collar-sized housing. The installation errors are then determined by the same rotational method used in the dynamic measurement: the probe is mounted on a calibrated standard high-precision three-axis turntable, which is a metrological instrument traceable to an angular standard and has an angular accuracy of ±0.01°, set at a well inclination of approximately 90°, and measurements are acquired at eight different tool-face angles during rotation. The installation-error angles *β* and *γ* are solved from multivariate linear regression and written into the firmware of the probe.

After the installation-error parameters were stored in the firmware, an independent inclination verification was carried out at several different well inclinations, which were different from the calibration orientation (approximately 90°). The reference inclination is provided by the turntable, i.e., an independently calibrated reference, rather than the output of the proposed method. The original calibration record is shown in Table 4.

At all tested well inclinations, the inclination error relative to the independently calibrated turntable reference is less than 0.1°. This verifies the reliability of the installation-error calibration and demonstrates that the probe can self-calibrate its own inclination measurement using its rotational data.

### 3.3. Dynamic Well Inclination Testing and Analysis

**Figure 8:**
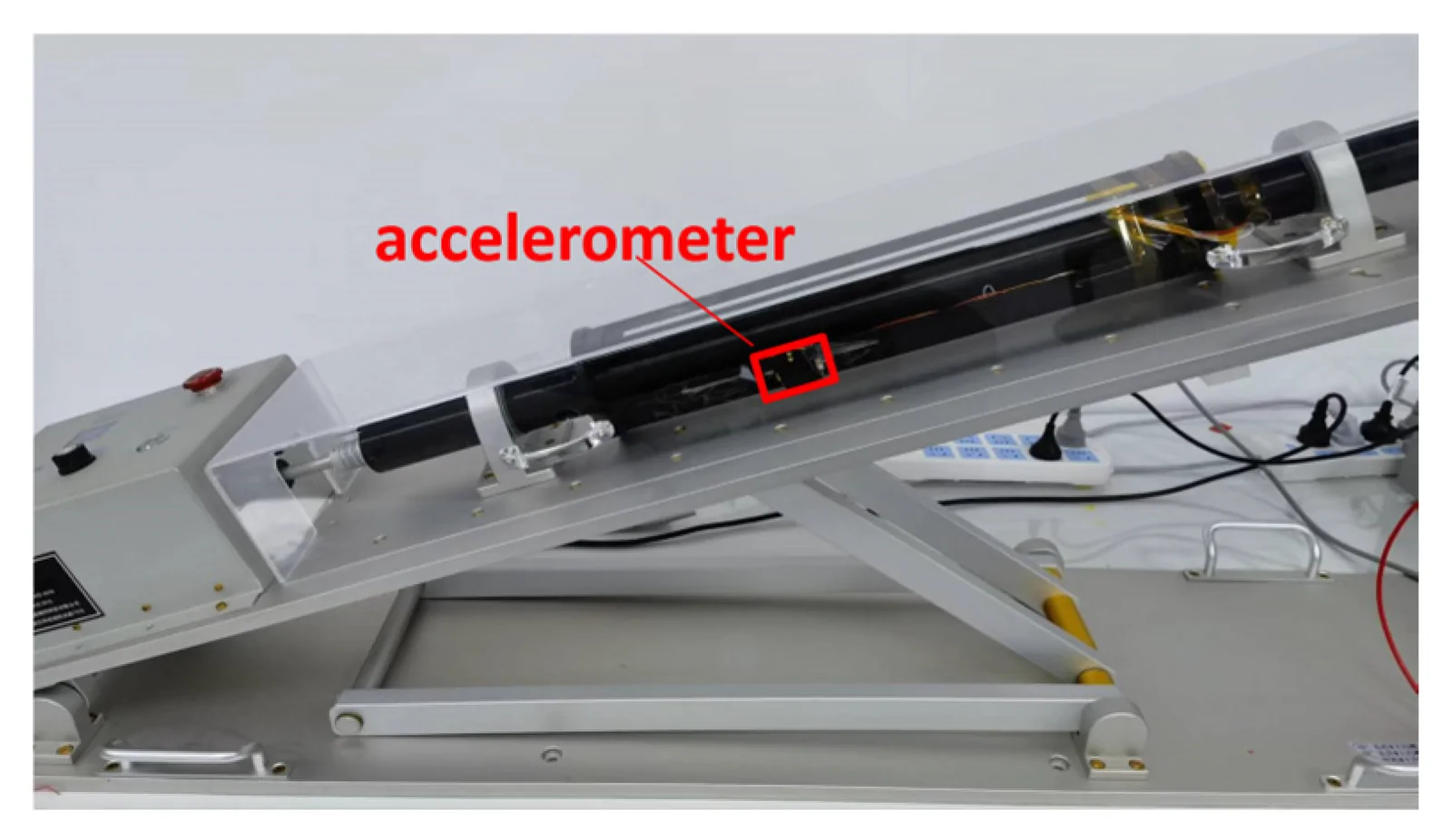
Rotational testing of well inclination measurement module.

The well inclination measurement module was installed on a rotational test turntable and the module was installed eccentrically on the rotating shaft ribs as shown in Figure 8. Three-axis accelerations were collected at seven well inclinations from 5.36° to 89.64° at static and at 30 r/min, 60 r/min, 100 r/min, and 150 r/min rotational speeds. Accelerations were collected for one revolution at eight different tool-face angles in the static case and at a sampling frequency of 500 Hz in the rotational condition.

**Figure 9:**
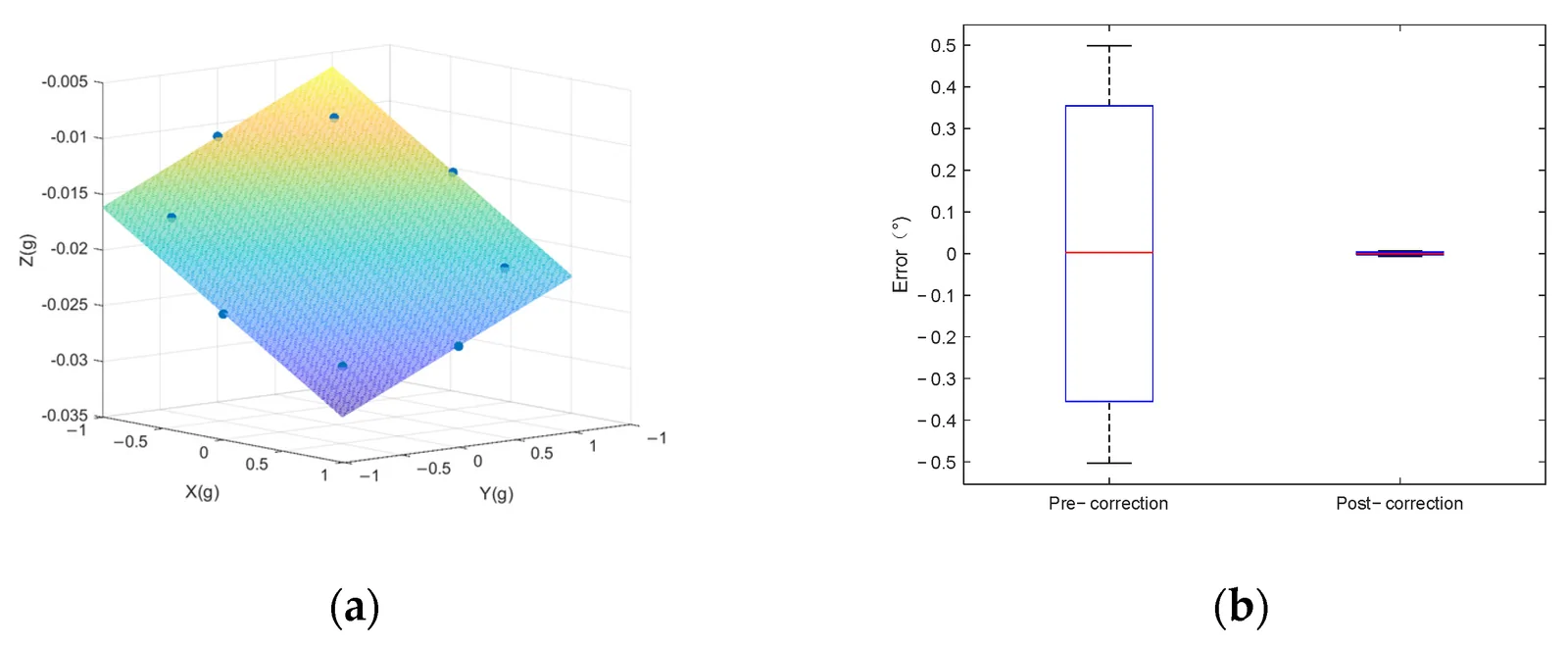
Calibration installation error at about 90° static well inclination. (**a**) Multivariate linear fitting model at about 90° static well inclination. (**b**) Well inclination measurement error before and after calibration installation error.

**Figure 10:**
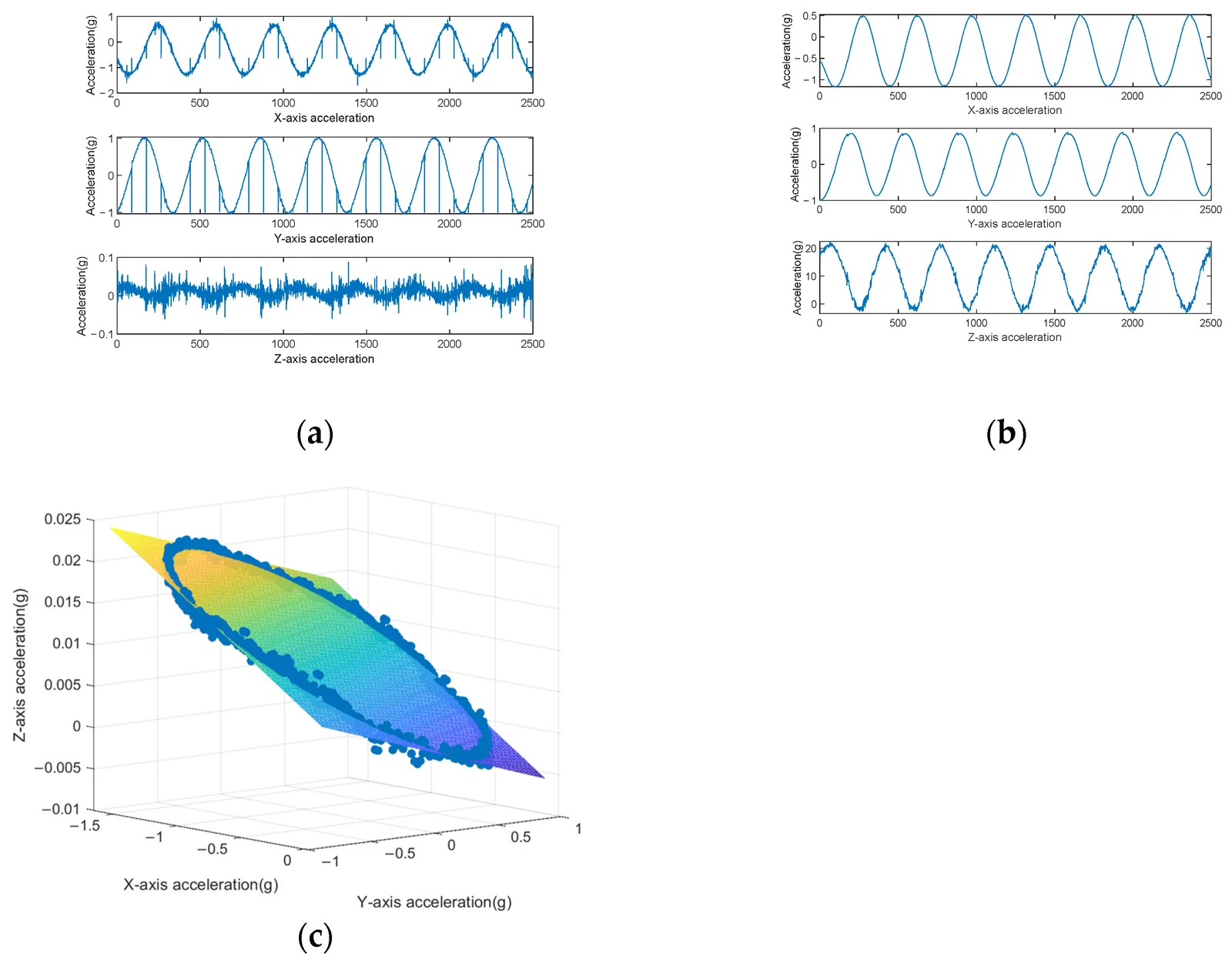
Dynamic inclination measurement at about 90° well inclination with a rotational speed of 100 r/min. (**a**) Triaxial acceleration. (**b**) Filtered triaxial acceleration. (**c**) Multivariate linear regression.

First, a multivariate linear fit to the acceleration tested in the static condition was performed to solve for the actual well inclination α and installation-error angles *β*, *γ*. The result of the multivariate linear fit to the triaxial acceleration for a static well inclination of about 90° is shown in Figure 9a; the actual well inclination is 89.64°. The comparison of well inclination error before and after the calibration of installation error is shown in Figure 9b. The maximum well inclination error decreases from 0.71° to 0.01°. Then, we conducted dynamic well inclination measurements. The Kalman filter is used to remove the noise from the data collected in the rotating case. The three-axis acceleration before filtering is shown in Figure 10a, and the three-axis acceleration after filtering is shown in Figure 10b. It can be observed that the Kalman filter effectively eliminates acceleration vibration noise. However, Z-axis acceleration still exhibits a sinusoidal waveform due to installation errors. The average value of the Z-axis acceleration over one rotation cycle is 9.9 mg. Without the influence of centrifugal force, the expected average Z-axis acceleration at this inclination angle should be 6.3 mg. Therefore, it is necessary to remove the centrifugal acceleration of the Z axis utilizing an algorithm to improve the accuracy of well inclination calculation. Then, multivariate linear regression is used to fit the three-axis acceleration, and the three-axis acceleration at a 90° well inclination at 100 r/min is shown in Figure 10c. The fitted triaxial-acceleration plane is centered among all the collected points, so the multivariate linear regression also has the effect of smoothing filtering, which can further improve the accuracy of dynamic well inclination measurement. Finally, the well inclination angle and installation error are derived by fitting the model with multivariate linear regression. This static value was independently verified against the calibrated turntable reference with an error below 0.1° (Section 3.2).

The static inclination, after correction with the installation errors stored in the firmware, is used as the reference for the dynamic measurements, and the calculation results are shown in Table 5. At the lowest tested inclination of 5.36°, where the horizontal gravity component is small, the difference between the static and dynamic estimates remains within ±0.18° at rotational speeds of 30, 60, 100, and 150 r/min. The well inclination error is less than ±0.18° and the installation angle errors γ, β are less than 0.002 rad.

The reason is that the rotational test turntable cannot provide an independently calibrated angular reference at the same accuracy level as the three-axis calibration turntable. The three-axis turntable presented in Section 3.2 is a metrological instrument that can be periodically calibrated and is traceable to an angular standard, with an angular accuracy of ±0.01°. However, it cannot accommodate the eccentric side-wall installation and continuous rotation required for the dynamic tests. Conversely, the rotational turntable can reproduce the eccentric installation and continuous rotation of the probe, but it is not a metrological instrument and does not provide a traceable angular reference.

Therefore, the dynamic results are compared with the static inclination obtained from the same probe, which was independently verified against the three-axis turntable with an error below 0.1° (Table 4). The absolute accuracy of the static calibration is reported separately in Section 3.2, and the dynamic results in Table 5 are presented as the difference from this independently verified static reference. At all tested inclinations and rotational speeds, the difference between the static and dynamic estimates is within ±0.18°, and the installation angle errors *β* and *γ* are less than 0.002 rad.

The error column reports the difference between the static and dynamic estimates, i.e., the static–dynamic consistency, rather than the absolute accuracy of the dynamic measurements.

**Figure 11:**
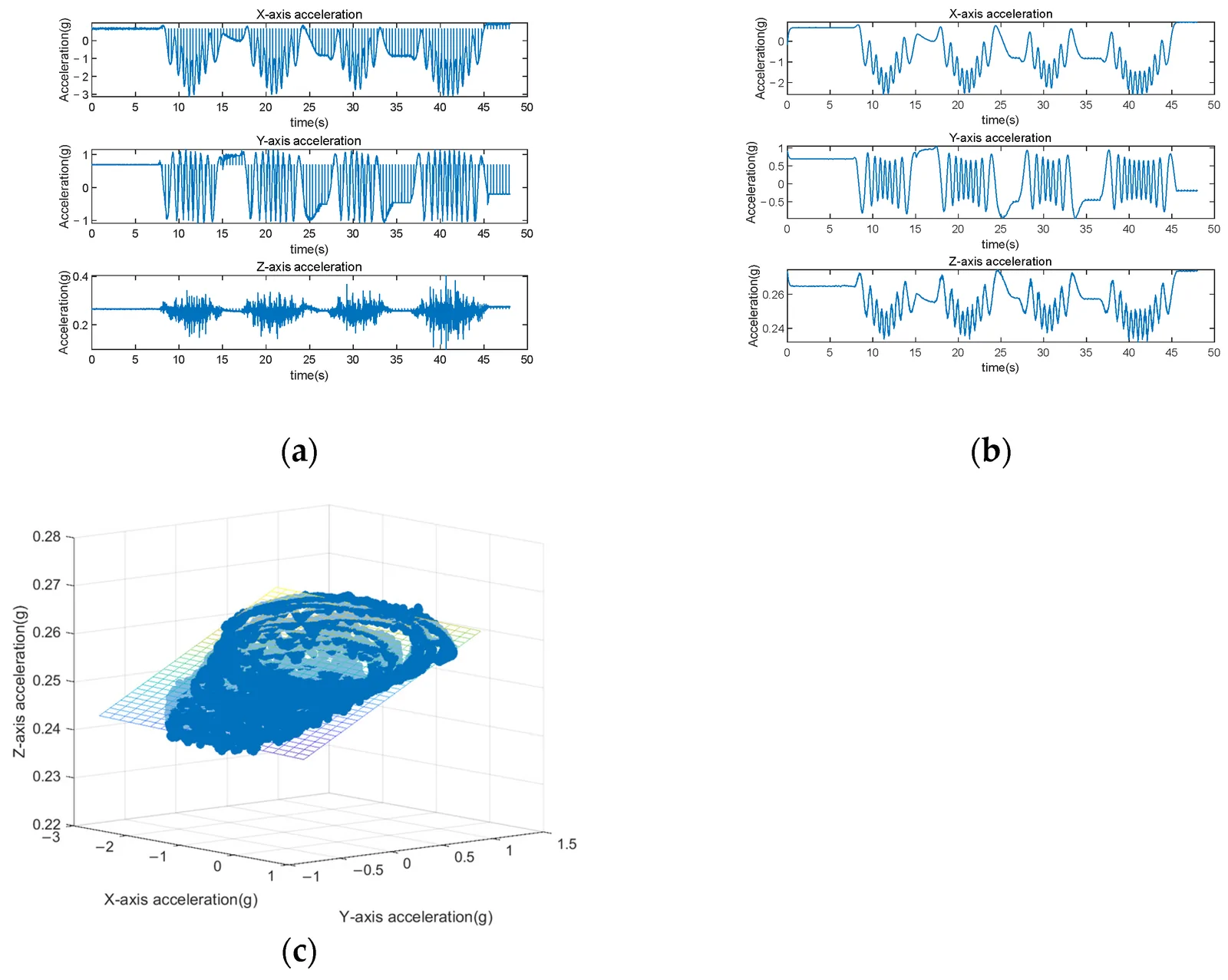
A variable-speed test at 75° inclination. (**a**) Triaxial acceleration. (**b**) Filtered triaxial acceleration. (**c**) Multivariate linear regression.

In addition to the constant-speed tests, a variable-speed test was carried out to simulate the rotational-speed fluctuation under downhole conditions. The rotational speed was increased gradually from 0 to 200 r/min, held for a range of 1–2 s, and then reduced to 0. The measured triaxial accelerations are shown in Figure 11. The results show that the method remains stable during speed changes, because the sliding regression window contains at least one complete rotation and the state classifier adapts to the speed variation.

A variable-speed test at 75° inclination was also performed on the rotational test turntable, and the results are shown in Figure 11. The static inclination solved at this condition is 74.56°, and the dynamic inclination is 74.43°, giving a difference of 0.13°. The linear regression still fits the data well. The method remains stable during speed changes, as shown by the regression fitting results in Figure 11.

It should be noted that a dedicated strong-vibration rotational test could not be carried out in this study, because the available test facility cannot apply vibration and rotation simultaneously. We plan to build such a facility and to perform systematic vibration tests in future work.

### 3.4. Comparison of Filtering and Self-Calibration Configurations

To separate the contributions of filtering and self-calibration, a simulation was carried out in which the installation errors were varied at each rotation cycle to represent installation-error changes after reinstallation. The true inclination was 40°, the rotation speed was 90 r/min, the eccentric distance was 0.15 m, and the centrifugal acceleration was *ω*^2^*r* = 13.3 m/s^2^ (about 1.33 g). Six rotation cycles were simulated, with 1000 samples per cycle. The installation errors were varied at each cycle: γ from 0.3° to 0.8°, β from 0.4° to 0.9°, and δ from 0.5° to 0.75°. Gaussian noise with a standard deviation of 0.5 g (X and Y axes) and 0.025 g (Z axis) was added to the raw acceleration.

Three configurations were compared on the same data: (1) filtering only: Kalman filtering is applied, and the inclination is calculated directly from the filtered triaxial acceleration without installation-error compensation. (2) Filtering with fixed installation-error compensation: a fixed set of installation-error angles (calibrated once at the first cycle) is used, and the inclination is obtained from the Z-axis component alone. (3) Complete online self-calibration: the installation-error angles are estimated online through multivariate linear regression and updated once per complete rotation.

**Figure 12:**
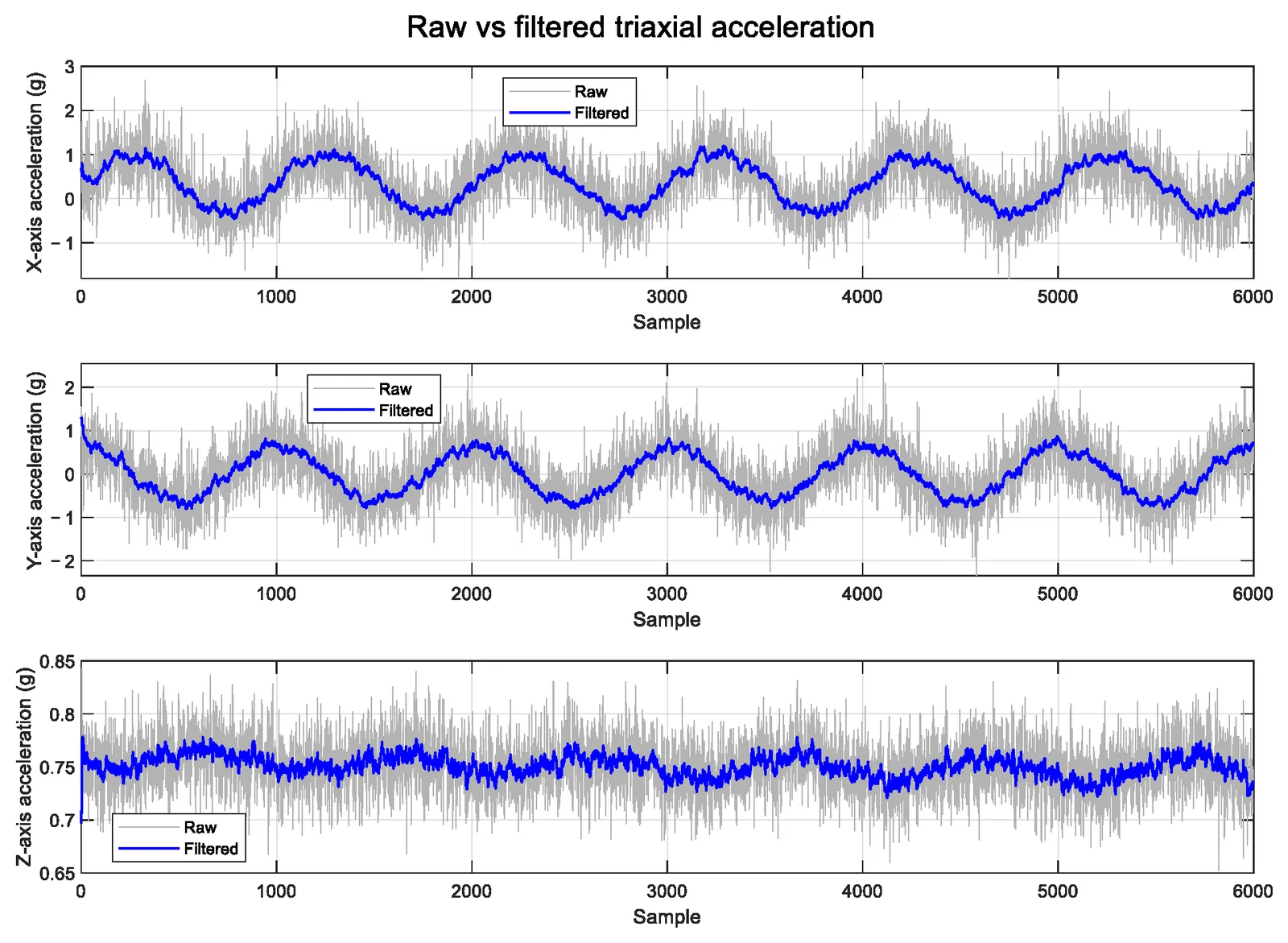
Raw and filtered triaxial acceleration results.

**Figure 13:**
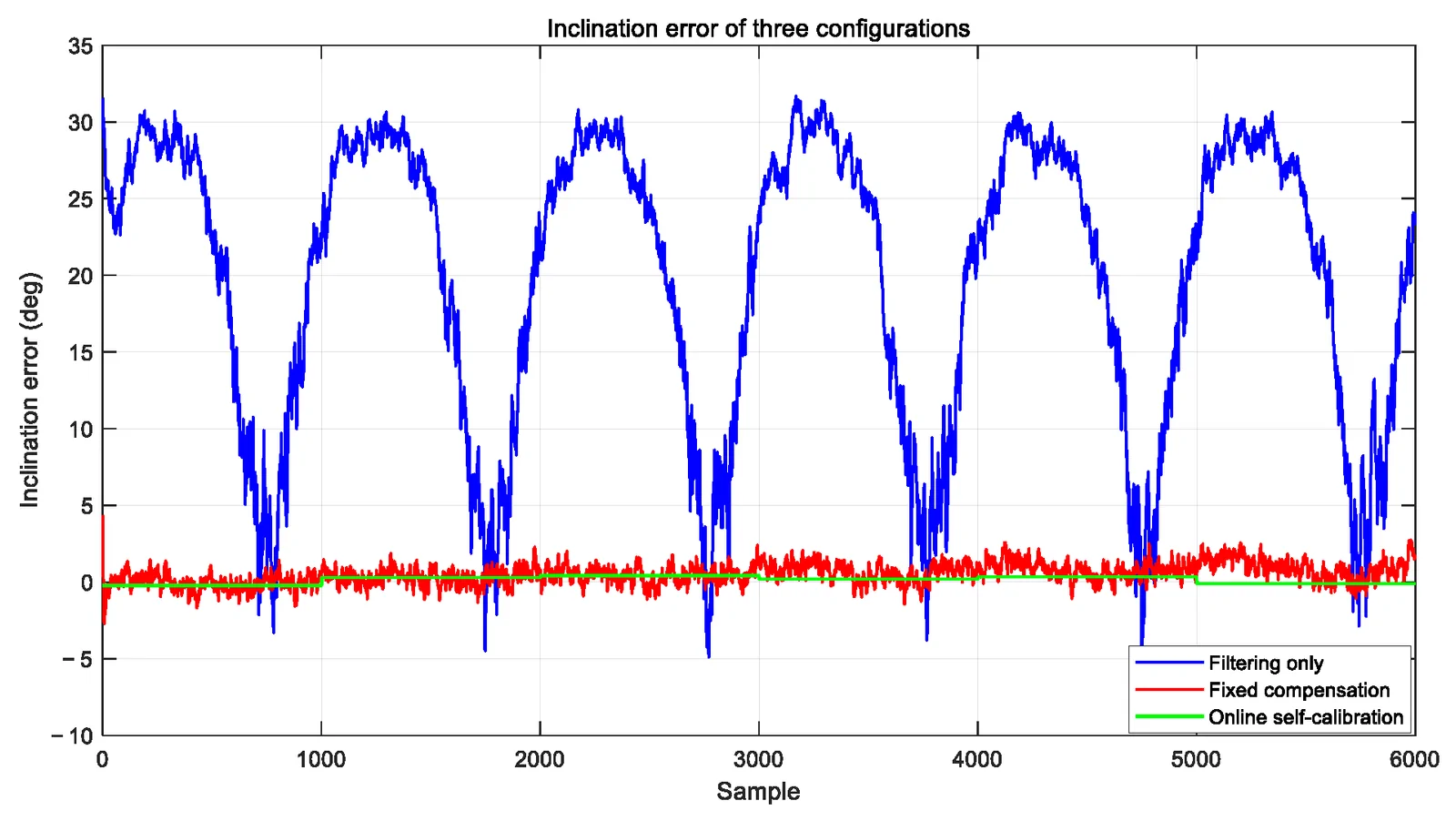
Inclination errors of the three configurations during the simulation.

Figure 12 shows the raw and filtered triaxial acceleration values. The filtered curves are smoother while the sinusoidal waveform caused by rotation is preserved. Figure 13 shows the inclination error of the three configurations over the simulation. The results are summarized in Table 6.

Filtering alone gives an MAE of 19.97°, an RMSE of 21.91°, and a maximum error of 30.17°, because the centrifugal term ω^2^r is a deterministic rotation-synchronous component that cannot be removed by filtering. Fixed compensation reduces the MAE to 0.59° and the RMSE to 0.75°, but the maximum error remains 2.24° because the installation errors change at each cycle and the fixed values become outdated. The complete online self-calibration reduces the MAE to 0.10°, the RMSE to 0.12°, and the maximum error to 0.21°, because the installation errors are re-estimated from the rotational data at each cycle. This comparison directly demonstrates the specific benefit of updating the installation errors online.

### 3.5. Field Test

**Figure 14:**
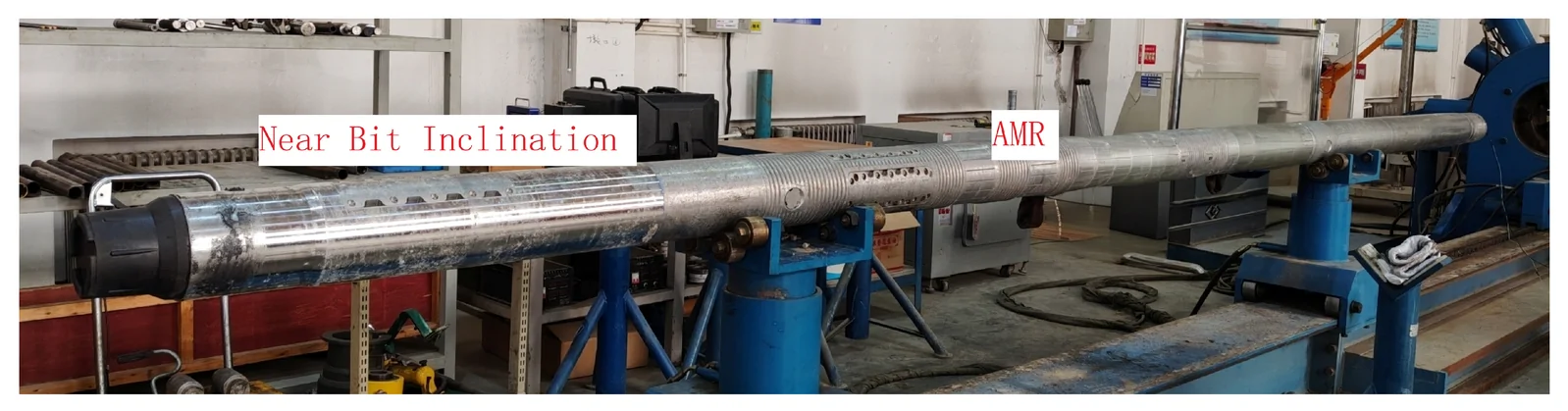
Installation configuration of the near-bit inclination tool and AMR.

**Figure 15:**
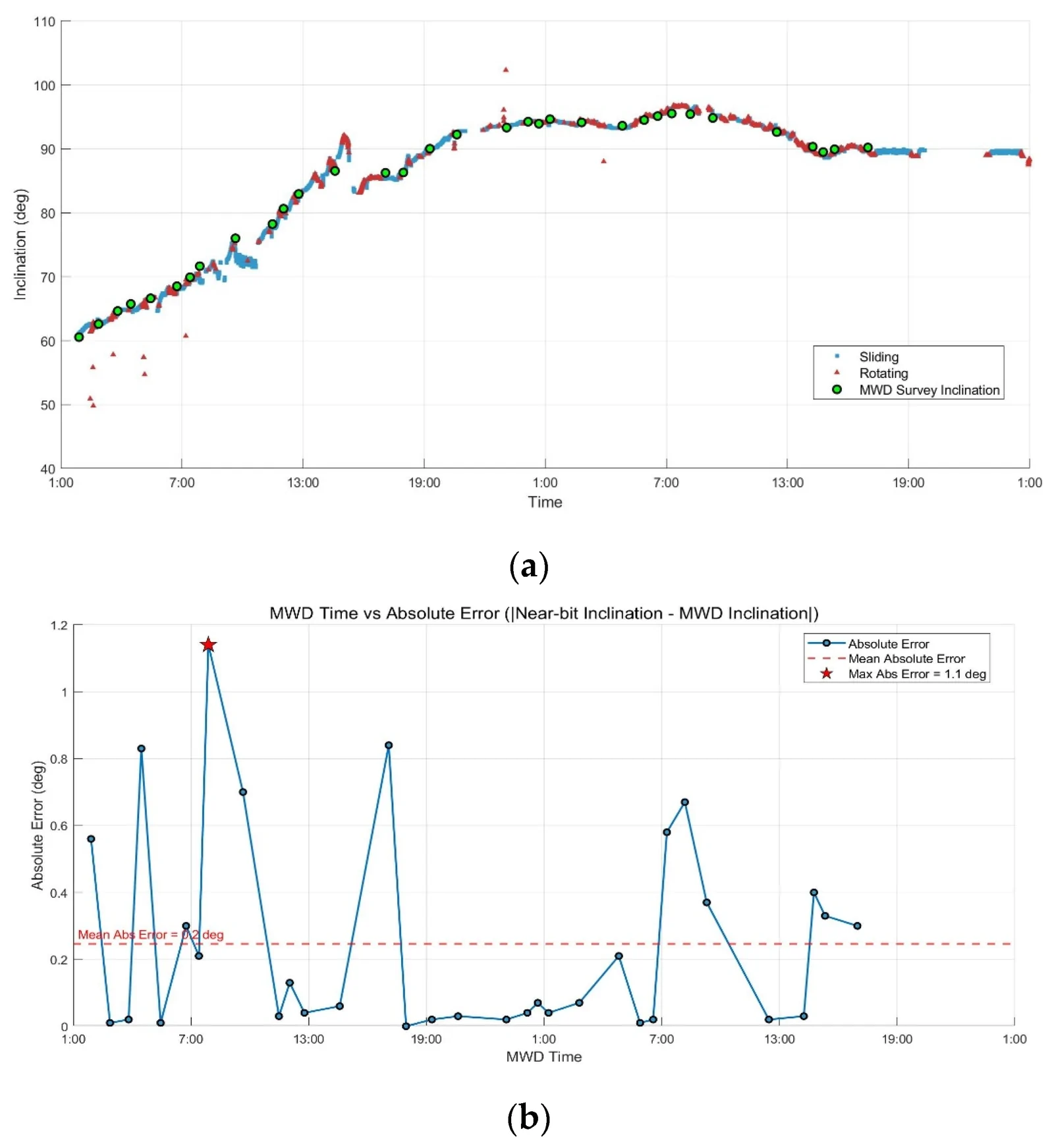
Comparison between near-bit inclination and survey measurements. (**a**) Near-bit inclination and MWD survey inclination versus time. (**b**) Absolute error at each matched measurement point.

The near-bit module was installed below the Azimuthal Multiple Resistivity (AMR) tool, as shown in Figure 14, which illustrates the installation configuration of the near-bit inclination tool and the AMR. This module enables well inclination measurement up to about 10 m ahead of the MWD, significantly facilitating the work of directional drillers on site. The MWD survey has an accuracy of 0.1°. Each near-bit inclination measurement carries a time stamp. The depth is determined from the time stamp, and the MWD data at the corresponding depth are mapped onto that time stamp, so that the near-bit and MWD measurements are compared at the same time point. A comparison between the near-bit inclination and MWD measurements at the same depths is presented in Figure 15, demonstrating that most of the near-bit data points follow the same trend as the MWD data. The measured inclination exhibits good consistency between rotary drilling and sliding drilling modes. With the near-bit inclination data, field engineers can perceive inclination changes ahead of time and achieve more accurate directional drilling. A total of 33 matched near-bit/MWD measurement pairs were obtained. The MWD reference accuracy is 0.1°, the mean absolute error is 0.2°, and the maximum absolute error is 1.1°. The absolute error at each matched point is plotted in Figure 15b.

**Figure 16:**
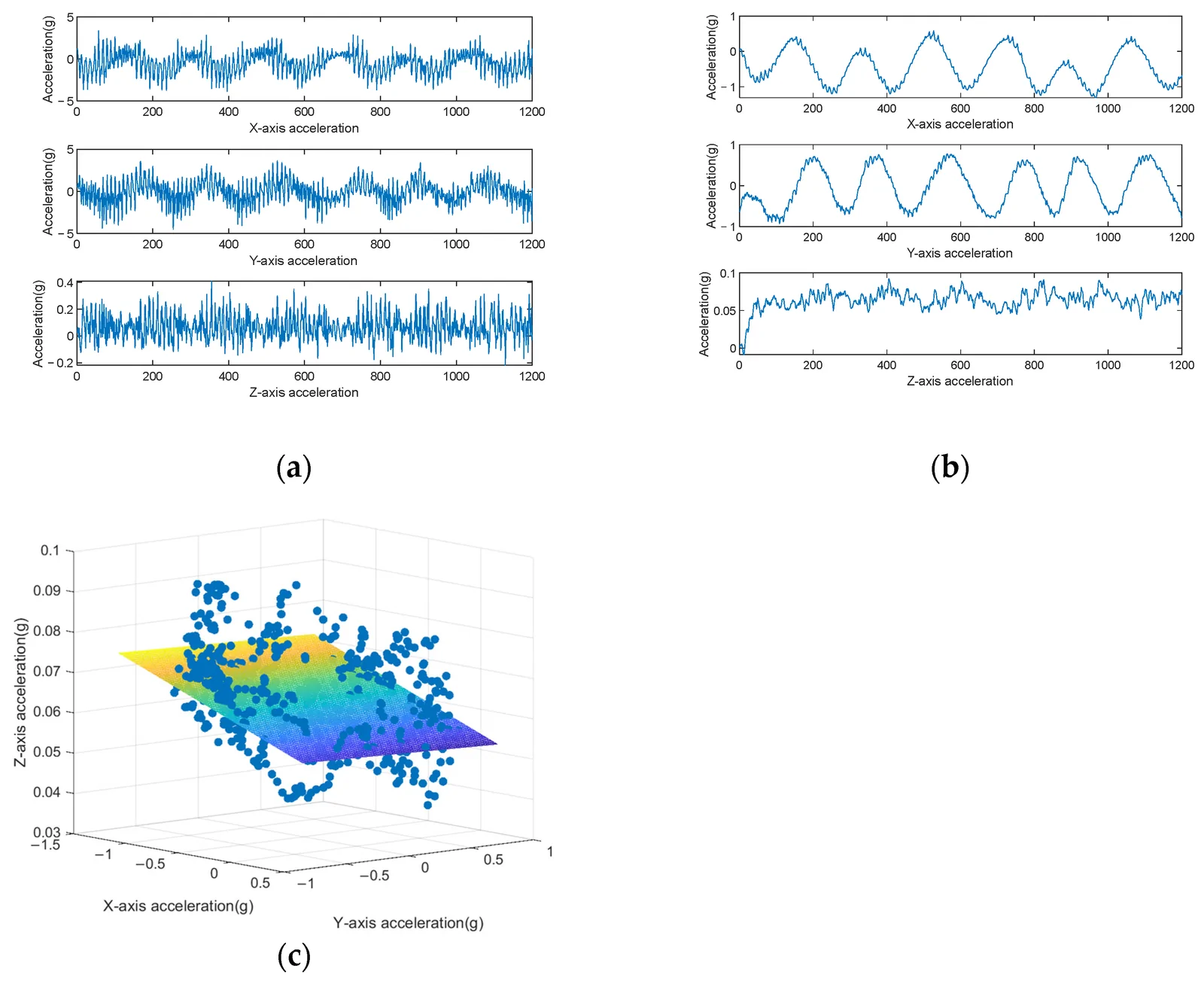
Dynamic inclination measurement model at field test. (**a**) Downhole raw triaxial-acceleration data. (**b**) Filtered triaxial acceleration. (**c**) Multivariate linear regression.

The real-time triaxial vibration acceleration over a certain period was analyzed. The downhole actual triaxial acceleration is shown in Figure 16a, and the filtered triaxial acceleration is presented in Figure 16b. The multivariate linear regression fitting model for downhole triaxial acceleration is displayed in Figure 16c. Based on this analysis, the calculated inclination was 93.5°, with an error of 0.2° compared to the MWD survey measurement of 93.3° at the same depth. Compared with laboratory test curves, the X-axis acceleration exhibits an offset from its normal equilibrium position (zero) due to centrifugal acceleration. However, downhole conditions introduce significantly more vibration noise, and the sinusoidal waveform caused by rotation becomes irregular with varying amplitudes. The larger deviations in the field data may be caused by strong downhole vibration, which reduces the signal-to-noise ratio of the acceleration signal, and by drill-string sag, i.e., the gravity-induced bending of the drill string, which makes the sensor orientation deviate from the borehole axis. These are identified as possible causes, since a dedicated strong-vibration rotational test could not be carried out in this study. It can be seen that the method achieves self-calibration of installation errors and enables reliable static and dynamic inclination measurements.

## 4. Conclusions

In this paper, a triaxial accelerometer-based well inclination measurement module is designed using a single-axis quartz flexure accelerometer and a dual-axis MEMS accelerometer. This design achieves the required measurement accuracy while reducing size and cost. A hybrid calibration method combining the ellipsoid method and the Levenberg–Marquardt method is proposed. The ellipsoid method is first used to initially calibrate the scale factor and zero bias, followed by the Levenberg–Marquardt method to further calibrate the scale factor, zero bias, and non-orthogonal angle. The resulting error in the sum of the three-axis acceleration vectors is less than 2 × 10^−3^ g, improving calibration accuracy and efficiency.

A static and dynamic well inclination self-calibration method based on installation-error compensation is proposed. A Kalman filter is employed to remove noise during drill collar rotation. A multivariate linear regression model is then applied to fit the three-axis acceleration data, enabling simultaneous determination of the true well inclination and installation errors while effectively mitigating the effects of centrifugal acceleration. The installation-error matrix is updated and stored during rotation and subsequently used for static condition measurements, eliminating the influence of installation errors on well inclination accuracy.

The installation-error calibration was independently verified against a calibrated standard high-precision three-axis turntable (±0.01°). At nominal inclinations of 0°, 3°, 10°, 30°, 45°, and 90°, the inclination error relative to the turntable reference is less than 0.1°. In the rotational tests, covering inclinations ranging from 5.36° to 89.64° and rotational speeds in the range of 30–150 r/min, the difference between the static and dynamic estimates is within ±0.18°. Since the rotational test turntable is not a metrological instrument and cannot provide a traceable angular reference, this result is reported as static–dynamic consistency, while the absolute accuracy of the static calibration (below 0.1°) is verified against the three-axis turntable. A variable-speed test (0–200 r/min) and a stick–slip simulation at 75° inclination further confirm that the method remains stable under rotational-speed fluctuations.

Field test results show that the near-bit inclination follows the same trend as the MWD survey, with a mean absolute error of about 0.2°. The larger deviations may be caused by strong downhole vibration, which reduces the signal-to-noise ratio of the acceleration signal, and by drill-string sag, i.e., the gravity-induced bending of the drill string. A dedicated strong-vibration rotational test could not be carried out in this study because the available test facility could not apply vibration and rotation simultaneously; this will be addressed in future work. The proposed approach achieves self-calibration of installation errors and addresses the challenge of installation-error variations that may occur during installation, storage, transportation, and field operation. The conclusions of this study are limited to the conditions actually tested: inclinations ranging from 5.36° to 89.64°, rotational speeds ranging from 30 to 150 r/min, and the field conditions described in Section 3.5. Combined strong-vibration and rotation tests were not performed, and the corresponding conclusions are not claimed.

## Figures and Tables

**Table 1:** Triaxial-acceleration voltages for different well inclinations and tool-face angles.

Inclination (°)	Tool Face (°)	X-Axis Voltage (V)	Y-Axis Voltage (V)	Z-Axis Voltage (V)
90	0	1.750585	1.474032	1.5661
90	45	1.695287	0.837117	1.573042
90	90	1.564565	0.581471	1.582391
90	135	1.435808	0.854863	1.575431
90	180	1.384125	1.501029	1.569827
90	225	1.43864	2.13471	1.561438
90	270	1.570242	2.394838	1.553968
90	315	1.699084	2.120841	1.550036
45	0	1.69577	1.430607	2.163612
45	90	1.575548	2.122214	2.1549
45	180	1.437921	1.516908	2.166036
45	270	1.550751	0.850016	2.171574
135	0	1.437479	1.483882	0.968047
135	90	1.565668	2.134928	0.957408
135	180	1.701301	1.492027	0.978198
135	270	1.571601	0.846508	0.975442
0	0	1.569883	1.492894	0.715574
180	0	1.56642	1.480767	2.409159

**Table 2:** The parameters calibrated by the ellipsoid and LM methods.

Parameters	Ellipsoid Method	Hybrid Ellipsoid and LM Method
*K_xx_*	5.451269623	5.451044472
*K_yy_*	1.102164	1.102800326
*K_zz_*	1.180115	1.180481148
*K_xy_*	0	−0.000453174
*K_xz_*	0	−0.000453174
*K_yx_*	0	0.000203413
*K_yz_*	0	0.010134825
*K_zx_*	0	0.035718856
*K_zy_*	0	0.010843032

**Table 3:** Quantitative comparison of calibration methods.

Method	Mean Error (10^−3^ *g*)	RMSE (10^−3^ *g*)	Maximum Absolute Error (10^−3^ *g*)
Ellipsoid only	0.038	5.04	10.39
Hybrid ellipsoid–LM	0.172	0.68	1.31

**Table 4:** Independent inclination verification against the turntable reference.

Azimuth Setting (°)	Nominal Inclination (°)	Tool-Face Setting (°)	Measured Inclination (°)	Maximum Absolute Error (°)
0	0	0	0.063	0.063
0	0	90	0.056	
0	0	180	0.023	
0	0	270	0.055	
0	3	0	3.043	0.083
0	3	90	3.059	
0	3	180	2.944	
0	3	270	2.917	
0	10	0	10.062	0.062
90	10	0	10.046	
180	10	0	10.027	
270	10	0	10.036	
0	30	0	30.030	0.030
90	30	0	30.016	
180	30	0	29.991	
270	30	0	30.005	
0	45	0	45.008	0.027
90	45	0	45.000	
180	45	0	44.973	
270	45	0	44.991	
0	90	0	89.969	0.078
0	90	90	90.062	
0	90	180	90.078	
0	90	270	89.984	
90	90	90	89.954	0.065
90	90	90	90.044	
90	90	90	90.065	
90	90	90	89.966	

**Table 5:** Results for different well inclination and rotational speeds.

Number	Rotation Speed (r/min)	Calculated Well Inclination (°)	*β* (rad)	*γ* (rad)	Error (°)
1	static	89.64	0.01201	0.00479	
	30	89.65	0.01218	0.00523	0.01
	60	89.68	0.01158	0.00553	0.04
	100	89.60	0.01124	0.00524	−0.04
	150	89.64	0.01105	0.00518	0.00
2	static	74.64	0.01183	0.00459	
	30	74.65	0.01165	0.00471	0.01
	60	74.67	0.01154	0.00476	0.03
	100	74.72	0.01118	0.00428	0.08
	150	74.68	0.01075	0.00500	0.04
3	static	59.65	0.01187	0.00461	
	30	59.72	0.01176	0.00501	0.07
	60	59.75	0.01146	0.00509	0.10
	100	59.67	0.01099	0.00466	0.02
	150	59.62	0.01036	0.00475	−0.03
4	static	44.66	0.01174	0.00440	
	30	44.83	0.01135	0.00473	0.17
	60	44.80	0.01129	0.00464	0.14
	100	44.83	0.01051	0.00507	0.17
	150	44.79	0.01035	0.00532	0.13
5	static	30.15	0.01203	0.00413	
	30	30.01	0.01215	0.00491	−0.14
	60	30.13	0.01189	0.00427	−0.02
	100	30.24	0.01176	0.00485	0.09
	150	29.98	0.01147	0.00501	−0.17
6	static	15.82	0.01095	0.00441	
	30	15.93	0.01161	0.00465	0.11
	60	15.76	0.01169	0.00476	−0.06
	100	15.67	0.01123	0.00493	−0.15
	150	15.88	0.01087	0.00592	0.06
7	static	5.36	0.01063	0.00469	
	30	5.21	0.01249	0.00679	−0.15
	60	5.18	0.01104	0.00527	−0.18
	100	5.23	0.01328	0.00426	−0.13
	150	5.22	0.01027	0.00378	−0.14

**Table 6:** Comparison of filtering and self-calibration configurations.

Configuration	MAE (°)	RMSE (°)	Maximum Error (°)
Filtering only	19.97	21.91	30.17
Filtering + fixed compensation	0.59	0.75	2.24
Complete online self-calibration	0.10	0.12	0.21

## Data Availability

The original contributions presented in this study are included in the article. Further inquiries can be directed to the corresponding author.
